# eGFR_cystatinC_/eGFR_creatinine_ ratio < 0.6 in patients with SARS-CoV-2 pneumonia: a prospective cohort study

**DOI:** 10.1186/s12882-023-03315-x

**Published:** 2023-09-13

**Authors:** Lauris Avotins, Juta Kroica, Aivars Petersons, Dace Zentina, Zaiga Kravale, Anna Saulite, Karlis Racenis

**Affiliations:** 1 Rīga Stradiņš University, Riga, Latvia; 2 Pauls Stradins Clinical University Hospital, Riga, Latvia

**Keywords:** Acute kidney injury, Cystatin C, Glomerular filtration rate, SARS-CoV-2 infection, Shrunken pore syndrome

## Abstract

**Background:**

Shrunken Pore Syndrome (SPS), defined as a reduced ratio between two estimated filtration rates (based on cystatin C and creatinine) is an increasingly recognized risk factor for long-term mortality. Although some patients with other conditions might be erroneously identified as SPS. Our aim was to bring the focus on possible pathophysiologic mechanisms influencing the ratio in the setting of SARS-CoV-2 pneumonia and acute kidney injury.

**Methods:**

A single-centered prospective cohort study was conducted to investigate biomarkers in symptomatic COVID-19 pneumonia patients admitted to a hospital in Latvia. Nineteen biomarkers were measured in blood and three in urine samples. Associations were sought between these biomarkers, chronic diseases and the estimated GFR_cystatinC_/eGFR_creatinine_ ratio < 0.6, mortality rates, and acute kidney injury development. Data analysis was performed using SPSS Statistics, with significance set at p < 0.05.

**Results:**

We included 59 patients (average age 65.5 years, 45.8% female) admitted with COVID-19. Acute kidney injury occurred in 27.1%, and 25.4% died. Ratio < 0.6 was seen in 38.6%, associated with female sex, diabetes, hypothyroidism, and higher age. Ratio < 0.6 group had mortality notably higher − 40.9% vs. 16.2% and more cases of acute kidney injury (40.9% vs. 18.9%). Cystatin C showed strong associations with the ratio < 0.6 compared to creatinine. Urea levels and urea/creatinine ratio were higher in the ratio < 0.6 group. After excluding acute kidney injury patients, ratio < 0.6 remained associated with higher cystatin C and urea levels. Other biomarkers linked to a kidney injury as NGAL, and proteinuria did not differ.

**Conclusion:**

We prove that reduced ratio is common in hospitalized patients with SARS-CoV-2 pneumonia and is associated with increased mortality during hospitalization. Factors that influence this ratio are complex and, in addition to the possible shrinkage of pores, other conditions such as thickening of glomerular basal membrane, comorbidities, prerenal kidney failure and others may play an important role and should be addressed when diagnosing SPS. We highlight the need for additional diagnostic criteria for SPS and larger studies to better understand its implications in acute COVID-19 settings.

**Supplementary Information:**

The online version contains supplementary material available at 10.1186/s12882-023-03315-x.

## Background

An impaired kidney filtration leads to an increase in plasma concentration of commonly used serum markers of kidney function, such as creatinine and cystatin C. An increased amount of each individually has been linked to higher mortality rates in various populations and clinical settings [1]. However, recent studies have found that the decreased ratio of estimated glomerular filtration rates (eGFR) calculated based on cystatin C (eGFR_cystatinC_) and creatinine (eGFR_creatinine_) is an independent risk factor for a long-term mortality in the general elderly population [2, 3] and especially in patients with an underlying cardiovascular disease [4, 5]. In 2015 *Grubb et al.* described this phenomenon of the eGFR_cystatinC_/eGFR_creatinine_ ratio below 0.6 (low-GFR_cys_/GFR_cr_) and proposed the term Shrunken Pore Syndrome (SPS) explaining that smaller molecules such as creatinine (0.113 kDa) are filtrated more freely than larger molecules, for example cystatin C (13.3 kDa), resulting in the lower ratio [6]. Similar results by *Purde et al.* showed that in addition to cystatin C other hormonally active proteins of molecular weight between 3.5 and 66.5 kDa are selectively retained, implying their role in increased mortality [3, 7]. The prevalence of this phenomenon is found to be from 0.7 to 22.3% in various populations [2–7].

An ongoing debate on the underlying pathophysiological principles of low-GFR_cys_/GFR_cr_ crystalizes two possible mechanisms: a reduction in pore size [6] and a thickening of the glomerular basal membrane (GBM). *Öberg et al.* demonstrate that low-GFR_cys_/GFR_cr_ is strongly associated with the thickness of the GBM in patients with a diabetic kidney disease to whom the thickening of the GBM is a hallmark of the kidney damage [8].

Regardless of the unclear mechanisms that lead to the phenomenon of a changed quality of kidney filtration, the importance of low-GFR_cys_/GFR_cr_ reflects in the emerging large-scale studies on the subject, and only one of those studies has described SPS in SARS-CoV-2 infection [9].

In late 2019, the new SARS-CoV-2 virus emerged and since then at the end of 2022 there have been more than 640 million confirmed cases worldwide, including more than six million deaths. The clinical spectrum of SARS-CoV-2 infection varies from asymptomatic to life-threatening disease, affecting mainly the respiratory system. But especially high mortality rates related to SARS-CoV-2 pneumonia are due to development of acute kidney injury (AKI), which develops from 17.7 to 56.9% [10–13] in patients with pneumonia. The risk of death in patients with SARS-CoV-2 infection and AKI is 11 times higher [14].

Our aim in this study was to discover the importance of low-GFR_cys_/GFR_cr_ in settings of SARS-CoV-2 pneumonia, and possible association with AKI and mortality.

## Materials & methods

### Study design

A single-centered prospective cohort study was conducted at Pauls Stradins Clinical University Hospital in Riga, Latvia, in association with Rīga Stradiņš University. Nineteen biomarkers were measured in venous and arterial blood samples and 3 biomarkers in urine samples from patients admitted from March 2021 until May 2021 on the day of admission. Associations were sought between acquired parameters and eGFR_cystatinC_/eGFR_creatinine_ ratio, mortality rates and development of AKI. STROBE Checklist of cohort studies was carried out.

### Ethical approval

Patients were informed of study design and possible outcomes on a day of admission. Patients were asked to give written informed consent for participation. Only patients willing and able to provide their written informed consent to participate in the study were included. This study was approved by the Ethics Committee of Pauls Stradins Clinical University Hospital (2502221-10 L).

### Patient selection

All 94 patients hospitalized from 13th of March to 4th of May 2021 to the COVID-19 ward of Pauls Stradins Clinical University Hospital were considered for inclusion. Hypoxemic SARS-CoV-2 positive patients with COVID-19 pneumonia were prospectively included. COVID-19 infection was defined as the positive SARS-CoV-2 of the nasal/throat swab by the reverse transcriptase polymerase chain reaction (RT-PCR) assay tested by the local diagnostic laboratory. COVID-19 pneumonia was determined by CT scan. Patients with clinical findings or previously known underlying diseases suggesting other causes of significant hypoxemia (chronic obstructive pulmonary disease (COPD), acute myocardial infarction, acute cerebral infarction, severe anemia (Hemoglobin < 80 g/L)) were excluded. Patients with active solid or hematological malignancy, active autoimmune disease, usage of immunosuppressive agents prior to admission ((prednisone > 10 mg/day or equivalent) more than 4 weeks), solid organ transplant, pregnancy, end stage renal disease with dialysis were excluded in order to reduce potential sources of bias in evaluating inflammatory biomarkers. Malnutrition was excluded on adapted Malnutrition Screening Survey filled on admission (based on Body mass index and unintentional weight loss in previous months). 34 patients matched exclusion criteria or refused to participate in the study. One patient included in the study was missing cystatin C value so was later excluded. Patients were treated according to the local protocol that included supplemental oxygen, thromboprophylaxis with low molecular weight heparins, dexamethasone 6 mg daily, empiric antibiotics and remdesivir if indicated. Treatment did not include hydroxychloroquine, azithromycin, convalescent plasma, tocilizumab or other immunomodulators. None of the patients were vaccinated against SARS-CoV-2.

### Data collection and analysis

Venous, arterial and urine samples were collected on the day of admission to hospital and analyzed in Joint Laboratory of Pauls Stradins Clinical University Hospital, accredited by Signatory of the Multilateral Agreement of the European Cooperation of Accreditation (EA MLA), LVS EN ISO 15189:2013. Serum creatinine was analyzed using modification of the kinetic Jaffé reaction on Siemens Atellica CH 930 (Siemens Healthcare) or Roche Cobas Integra 400+ (Roche Diagnostics). Serum Cystatin C was analyzed using immunoturbidimetry method on Roche Cobas Integra 400+ (Roche Diagnostics). Estimated glomerular filtration rate (eGFR) was calculated based on serum creatinine (2021 CKD-EPI Creatinine) and Cystatin-C level (2021 CKD-EPI Cystatin C). Threshold of low-GFR_cys_/GFR_cr_ ratio was set < 0.6 [2, 6] measured during the first day of hospitalization. Information on comorbidities was extracted from the medical records. Chronic kidney disease (CKD) was defined as eGFR_creatinine_ < 60 mL/min in the year before hospitalization for COVID-19. AKI was defined according to the KDIGO guidelines as an increase in serum creatinine by 26.5 µmol/l within 48 h or an increase in serum creatinine 1.5 times baseline, which is known or presumed to have occurred within the prior 7 days. Baseline serum creatinine was determined from the laboratory database if available. For those lacking baseline creatinine, diagnosis of AKI was made based on clinical data- negative history of CKD, returning of serum creatinine levels to normal during hospitalization. None of the patients had their baseline cystatin C values. Ratio between pO_2_/FiO_2_ (P/F ratio) was calculated using pO_2_ measurement acquired after additional oxygen treatment if necessary for at least 30 min to achieve SpO2 > 94%.

Data were analyzed with IBM SPSS Statistics (release 27.0.1.0). The Mann-Whitney U test or Chi-square test were used to test differences between groups. Continuous data were summarized with mean and standard deviation or median and interquartile range (IQR) depending on distribution of values. Case-wise deletion was applied when addressing missing data. p value < 0.05 was considered significant.

## Results

Average age of the 59 patients included in the study was 65.5 years and 45.8% of patients were female (Table 1). Acute kidney injury (AKI) as a complication developed in 27.1% of cases, while 25.4% of patients died during the hospitalization. Most common comorbidities were arterial hypertension (71.2%), diabetes (35.6%), adiposity (35.6%) and congestive heart failure (33.9%). A total of 22 patients (38.6%) had low-GFR_cys_/GFR_cr_, with statistically significant prevalence of female sex (p = 0.008), diabetes (p = 0.019), hypothyroidism (p = 0.021) and higher age (p = 0.032) No significant age or chronic disease difference between survivors and non-survivors were noted (*Additional file 1*). Mortality was notably higher in the group with low-GFR_cys_/GFR_cr_ − 40.9% vs. 16.2% (p = 0.035). Low-GFR_cys_/GFR_cr_ patient’s chances of dying (OR) are 3.57 (95% CI 1.06–12.11) times higher than a patient with high-GFR_cys_/GFR_cr_ (p = 0.040). Although a difference in development of AKI between the two categories was not observed (p = 0.066), patients with low-GFR_cys_/GFR_cr_ had more cases of AKI than those with a ratio above 0.6 (40.9% and 18.9%, respectively). Six out of nine patients with AKI and low-GFR_cys_/GFR_cr_ died compared to no mortality in seven patients with AKI and high-GFR_cys_/GFR_cr_.

**Table 1:** Baseline demographics

Characteristics	eGFR_Cystatin C_/eGFR_Creatinine_		Overall (n = 59)	*p*, value
	< 0.6 (n = 22)	≥ 0.6 (n = 37)		
Death, n (%)	9 (40.9)	6 (16.2)	15 (25.4)	0.035
Sex (Female), n (%)	15 (68.2)	12 (32.4)	27 (45.8)	0.008
Age (years), mean (± SD)	70.4 (12.3)	62.6 (13.5)	65.5 (13.5)	0.032
eGFR_creatinine_ before admission, (mL/min/1.73m^2^), mean (± SD)	77.2 (22.3)	84.5 (19.8)	81.8 (20.9)	0.197
Comorbidities, n (%)				
Chronic kidney disease	5 (22.7)	5 (13.5)	10 (16.9)	0.362
Acute kidney injury during hospitalization	9 (40.9)	7 (18.9)	16 (27.1)	0.066
Diabetes	12 (54.5)	9 (24.3)	21 (35.6)	0.019
Arterial hypertension	14 (63.6)	28 (75.7)	42 (71.2)	0.323
Gout	1 (4.5)	2 (5.4)	3 (5.1)	0.884
Adiposity	10 (45.5)	11 (29.7)	21 (35.6)	0.233
Congestive heart failure	9 (40.9)	11 (29.7)	20 (33.9)	0.380
Gastritis	0 (0)	4 (10.8)	4 (6.8)	0.110
Atrial flutter	3 (13.6)	10 (27.0)	13 (22.0)	0.230
Liver cirrhosis	1 (4.5)	1 (2.7)	2 (3.4)	0.705
Hypothyroidism	3 (13.6)	0 (0)	3 (5.1)	0.021

eGFR_Creatinine_- estimated glomerular filtration rate (Creatinine)

Other clinically important markers were compared between the groups of low and high-GFRcys/GFRcr (Table 2). Our data shows no difference in serum creatinine and eGFR_creatinine_ values (p = 0.713 and p = 0.485, respectively), in contrast to strong association with cystatin C and eGFR_cystatinC_ values (p < 0.001) in both groups, indicating the importance of relatively high cystatin C when calculating the eGFR_cystatinC_/eGFR_creatinine_ ratio. Urea measured in patients’ serum was significantly higher in patients with low-GFR_cys_/GFR_cr_ (p = 0.020), hence a higher urea/creatinine ratio in the group (p < 0.001). It is important to note that other biomarkers linked to a kidney injury such as NGAL and proteinuria did not show a statistically significant difference.

**Table 2:** Parameter association with eGFR_Cystatin C_/eGFR_Creatinine_ in all patients

Parameter, median (IQR)	eGFR_Cystatin C_/eGFR_Creatinine_		Overall	*p*, value
	< 0.6	≥ 0.6		
pH	7.43 (0.06)	7.44 (0.07)	7.44 (0.06)	0.643
pCO_2,_*mmHg*	34.50 (8.00)	34.00 (5.00)	34.00 (6.00)	0.631
Na, *mmol/L*	136.00 (5.00)	133.00 (5.75)	134.00 (6.00)	0.039
K, *mmol/L*	3.90 (0.80)	3.90 (0.50)	3.90 (0.52)	0.730
Ca, *mmol/L*	1.10 (0.11)	1.08 (0.07)	1.09 (0.08)	0.217
Glu, *mmol/L*	9.30 (3.70)	8.00 (3.70)	8.65 (3.25)	0.239
Lac, *mmol/L*	1.20 (0.58)	1.00 (0.50)	1.00 (0.48)	0.191
HCT, *%*	38.00 (13.00)	39.00 (9.45)	38.00 (9.75)	0.209
FiO_2,_*%*	46.00 (40.00)	46.00 (29.00)	46.00 (32.00)	0.848
P/F ratio	131.00 (114.25)	165.00 (97.75)	151.00 (100.50)	0.274
Albuminuria, *mg/g*	5.47 (5.52)	7.29 (12.56)	6.44 (6.66)	0.139
NGAL, *ng/mL*	56.30 (81.35)	37.25 (45.80)	40.30 (53.05)	0.160
Urea, *mmol/L*	11.55 (7.35)	6.80 (4.32)	8.05 (7.00)	0.020
Cystatin C, *mg/L*	1.73 (0.83)	1.19 (0.48)	1.35 (0.72)	< 0.001
Creatinine, *µmol/L*	76.50 (42.50)	80.00 (29.00)	80.00 (32.00)	0.713
Urea/Creatinine ratio	126.23 (65.15)	82.57 (39.11)	101.76 (52.90)	< 0.001
eGFR Creatinine, *mL/min/1.73m*^*2*^	79.50 (36.75)	81.00 (28.50)	81.00 (35.00)	0.485
eGFR Cystatin C, *mL/min/1.73m*^*2*^	35.00 (24.50)	60.00 (34.50)	50.00 (38.00)	< 0.001
WBC, *× 10*^*9*^*/L*	6.35 (4.02)	5.80 (3.85)	6.00 (4.10)	0.944
Neu, *× 10*^*9*^*/L*	5.10 (3.60)	4.70 (3.30)	4.85 (3.40)	0.885
Ly, *× 10*^*9*^*/L*	0.75 (0.43)	0.80 (0.40)	0.80 (0.40)	0.847
LRG, *µg/mL*	70.12 (44.54)	87.29 (22.75)	85.97 (33.16)	0.045
Ferritin, *µg/L*	716.35 (956.38)	878.80 (882.40)	741.60 (919.10)	0.573
CRP, *mg/L*	83.19 (123.47)	107.61 (77.81)	100.51 (91.83)	0.331
PCT, *ng/mL*	0.12 (0.55)	0.15 (0.24)	0.14 (0.29)	0.363
IL-6, *pg/mL*	12.20 (58.95)	19.80 (56.90)	18.90 (55.90)	0.875

pCO_2_ - partial pressure of carbon dioxide; Na- serum sodium; K- serum potassium; Ca- ionized serum calcium; Glu- serum glucose; Lac- serum lactates; HCT- hematocrit; FiO_2_- fraction of inspired oxygen; P/F ratio- PaO_2_/FiO_2_ ratio; Albuminuria- urine albumin/urine creatinine ratio; NGAL- urine neutrophil gelatinase-associated lipocalin; Urea- serum urea; Cystatin C- serum Cystatin C; Creatinine- serum creatinine; eGFR Creatinine- estimated glomerular filtration rate (Creatinine); eGFR Cystatin C- estimated glomerular filtration rate (Cystatin C); WBC- absolute white blood cell count; Neu- absolute neutrophil count; Ly- absolute lymphocyte count; LRG- serum Leucine-rich Alpha-2 Glycoprotein; Ferritin- serum ferritin; CRP- serum C-reactive protein; PCT- serum procalcitonin; IL-6- serum interleukin-6

Since cystatin C is known as an early indicator of kidney injury, in further statistical analysis we excluded patients who developed AKI, thus limiting possible impact of AKI in low-GFR_cys_/GFR_cr_ (Table 3). As before, low-GFR_cys_/GFR_cr_ was calculated at the expense of significantly lower eGFR_cystatinC_ (p < 0.001), with urea and urea/creatinine ratio remaining higher also in this group (p = 0.039 and p = 0.019, respectively).

**Table 3:** Parameter association with eGFR_Cystatin C_/eGFR_Creatinine_ in patients without AKI.

Parameter, median (IQR)	eGFR_Cystatin C_/eGFR_Creatinine_		Overall (n = 43)	*p*, value
	< 0.6 (n = 13)	≥ 0.6 (n = 30)		
Death, n (%)	3 (23.1)	6 (20)	9 (20.9)	0.820
Albuminuria, *mg/g*	6.44 (6.35)	7.30 (7.08)	6.59 (6.62)	0.160
NGAL, *ng/mL*	34.90 (48.13)	35.15 (30.20)	35.15 (33.30)	0.636
Urea, *mmol/L*	9.80 (6.32)	6.25 (2.78)	6.55 (4.60)	0.039
Cystatin C, *mg/L*	1.54 (0.70)	1.10 (0.41)	1.20 (0.39)	< 0.001
Creatinine, *µmol/L*	71.00 (38.50)	77.00 (26.25)	77.00 (28.00)	0.483
Urea/Creatinine ratio	116. 68 (64.39)	82.47 (39.55)	97.07 (41.09)	0.019
eGFR Creatinine, *mL/min/1.73m*^*2*^	79.00 (40.25)	90.50 (25.25)	87.00 (26.00)	0.578
eGFR Cystatin C, *mL/min/1.73m*^*2*^	39.00 (24.00)	69.50 (28.25)	58.00 (33.00)	< 0.001

Albuminuria- urine albumin/urine creatinine ratio; NGAL- urine neutrophil gelatinase-associated lipocalin; Urea- serum urea; Cystatin C- serum Cystatin C; Creatinine- serum creatinine; eGFR Creatinine- estimated glomerular filtration rate (Creatinine); eGFR Cystatin C- estimated glomerular filtration rate (Cystatin C)

## Discussion

The goal of our study was to investigate the prevalence and associated factors of low-GFR_cys_/GFR_cr_ in patients admitted to a hospital with acute, symptomatic SARS-CoV-2 infection. When *Grubb et al.* first described Shrunken Pore Syndrome they gave rise to caution that some patients with other conditions might be erroneously identified with a SPS and suggested other additional diagnostic criteria. Other studies have underlined the same flaws [2, 3, 6]. We would like to address our concern that estimated GFR formulas up to date are developed for usage solely in chronic kidney disease. However, to justify the use of such formulas, we would like to give some clarifications.

First, other previously published study by *Larsson AO et al.* [9] has also used estimated GFR in critically ill COVID-19 patients to determine eGFR by cystatin C (Caucasian-Asian-Pediatric-Adult equation -CAPA), and Lund–Malmö creatinine equation (LMR) to calculate eGFR determined by creatinine. Neither of formulas have been approved for use in AKI. We feel obliged to do similarly, in order to produce a study compatible with research already published.

Second, Shrunken Pore Syndrome is defined by eGFR_cystatinC_/eGFR_creatinine_ ratio, thus acute kidney injury or unknown previous creatinine level would disable us using this ratio in research. So, in our study we use CKD-EPI formulas (for creatinine and cystatin C) as a tool to adjust measured creatinine and cystatin values by age and sex and calculate a ratio between two eGFRs. Obtained filtration rates are not used in clinical decision making, where, in fact, they poorly estimate filtration rates in acute settings. To clear some of the doubts we already analyzed a subgroup of patients without AKI (according to the KDIGO, not estimated GFR) (*see* Table 3). Data shows that in both groups (all patients and those without AKI) there are similar tendencies in results. Therefore, if we want to analyze SPS in acute settings, we do not have any other option than to either use eGFR or (less likely) include patients with known SPS, before developing acute illness. We tried to use the given definition of SPS in this setting to test for the factors that can explain the differences in eGFR calculations and see if this is solely associated with SPS.

Keeping this in mind we have retained ourselves from using the term Shrunken Pore Syndrome, instead of low-GFR_cys_/GFR_cr_.

Further on we will discuss various mechanisms and suggest possible explanations that are associated with low-GFR_cys_/GFR_cr_ in our cohort.

The studies carried out so far have mainly focused on outpatient cohorts, yielding results that the prevalence of this phenomenon ranged from 0.7 to 22.3% [2–7]. The only study so far with SARS-CoV-2 infected patients admitted to intensive care unit shows prevalence of 24% [9]. In our study 38.6% of patients had low-GFR_cys_/GFR_cr_, which is significantly higher than described before. It has been shown that low-GFR_cys_/GFR_cr_ is associated with female sex, thus one of the possible affecting factors could be the relatively high proportion of women (45.8%) in our study compared to other study, where the overall proportion of women participating in the study did not reach a third of all patients (28.1%), leading to a lower prevalence in the whole study. The general health of patients, as well as the age of the patient, could increase the prevalence as previously suggested by *Malmgren et al.* [2]. Elderly people and patients with chronic diseases are well known risk groups for symptomatic and more severe SARS-CoV-2 infection, therefore more likely to be admitted to hospital. Our results confirm that low-GFR_cys_/GFR_cr_ was detected more common between elderly, and chronic conditions as arterial hypertension, diabetes, and adiposity were more prevalent with low-GFR_cys_/GFR_cr_ than in the general population.

Most importantly in our study group, low-GFR_cys_/GFR_cr_ is associated with higher mortality rates. Mortality was notably higher in the group with low ratio − 40.9% vs. 16.2% and low-GFR_cys_/GFR_cr_ patient chances of dying are 3.57 times higher than a patient with high- GFR_cys_/GFR_cr_.

Since the ratio of estimated filtration rates is directly proportional to filtration rates based on cystatin C and creatinine, factors affecting serum concentrations of each- cystatin C and creatinine- should be looked at. Cystatin C is endogenously produced at a constant rate, freely filtered in the glomerulus, neither reabsorbed nor secreted in the renal tubule, nor extra renally eliminated [15]. Its plasma concentration reversibly increases in the third trimester of pregnancy together with other molecules of similar weight and different production mechanisms, supporting the theory of changes in the quality of filtration during pregnancy [16]. Plasma concentration of cystatin C increases in corticosteroid use [17–19], and cystatin C levels appear to be elevated in patients with hypothyroidism and decreased in those with hyperthyroidism, associating this with changes in baseline metabolism and thus changes in GFR [20].

The development of AKI in SARS-CoV-2 infection is multifactorial and is associated with tubular injury, systemic inflammation, and imbalanced coagulation; however, the role of endothelial dysfunction is also crucial [21–23]. The renal function biomarker such as cystatin C more precisely and more early reflect renal endothelial damage and are less affected by extrarenal and tubular influences [24]. Increased cystatin C with still normal creatinine and consequently a lower ratio could signal early renal damage. Similar studies have shown cystatin C increase in severely ill patients and that it can be used as a predictive marker for mortality in COVID-19 patients [25]. According to our results we would speculate that the criteria of AKI should be changed in favour to cystatin C because of underestimation of AKI using creatinine values (relative to cystatin C). In future other studies should also focus on usage of cystatin C level as AKI criteria as it has shown its usefulness [26]. Though it is unlikely that patients will have baseline cystatin C in large cohorts in near future.

In fact, acute kidney injury was observed more in the group with low-GFR_cys_/GFR_cr_ (40.9% and 18.9%, respectively). Interestingly, six out of nine patients with AKI and low-GFR_cys_/GFR_cr_ died compared to no mortality in seven patients with AKI and high-GFR_cys_/GFR_cr_, showing a tendency of higher mortality in patients with low-GFR_cys_/GFR_cr_.

Even though it seems that low-GFR_cys_/GFR_cr_ is calculated on behalf of increase in cystatin C, there are more factors that affect creatinine than cystatin C. Creatinine is a by-product of muscle metabolism, and its release into the circulation vary greatly with age (production decreases with older age, largely because of the reduction in muscle mass), gender (female sex tends to have less muscle mass than male), individual muscle mass, certain diseases and conditions leading to reduction of muscle mass (rhabdomyolysis, movement impairment) and diet [27]. Although we recognized and excluded patients with malnutrition, it is important to note that individual body or muscle mass is not considered when calculating eGFR_creatinine_. This results in a lower ratio for those included who do not yet have malnutrition but are underweight and are at increased risk for severe SARS-CoV-2 infection and death [28].

An alternate cause of low-GFR_cys_/GFR_cr_ to pore shrinkage is thickening of GBM that decrease the filtration of larger molecular weight molecules, proven in patients with diabetic kidney disease [8]. More than a third of the patients (35.6%) included in our study had diabetes, with a statistically important predominance in low-GFR_cys_/GFR_cr_ vs. high-GFR_cys_/GFR_cr_ (54.5% and 24.3%, respectively), similarly to the previously published study on SPS in COVID-19 patients. Diabetes is a known risk factor for severe SARS-CoV-2 infection and is associated with higher mortality rates [29]. Although there was no statistically significant association seen between diabetes and mortality in our study (p = 0.680), these results favor thickened GBM theory, and the role of diabetes should be addressed in further studies.

Low-GFR_cys_/GFR_cr_ is now recognized as an independent risk factor for long-term mortality in various populations and clinical settings and most of the authors acknowledge the lack of information on other renal biomarkers associated with low-GFR_cys_/GFR_cr_. We measured albuminuria as a marker of structural damage to the glomerulus, urinary NGAL indicating proximal tubular damage, where it should normally be reabsorbed, and serum urea, calculating the serum urea/creatinine ratio. In prerenal injury urea increases disproportionately to creatinine due to increased proximal tubular re-absorption. Neither albuminuria nor urinary NGAL were significantly higher in patients with low-GFR_cys_/GFR_cr_ excluding glomerular and proximal tubular damage as underlying pathophysiological mechanisms. In addition, proximal tubular function in these patients was not impaired as shown by an increase in the urea and urea/creatinine ratio in the group. Increased urea/creatinine ratio could predict overestimation of creatinine-based eGFR [30]. Overestimation is defined by relatively higher eGFR_creatinine_ than eGFR_cystatinC_, which, in fact, is similar with a definition of low-GFR_cys_/GFR_cr_, suggesting overlap of two entities and might be one of the errors, when making diagnosis of SPS.

To evaluate urea/creatinine ratio, we must analyze possible mechanisms that could increase serum urea levels.

- Higher catabolism in patients with low-GFR_cys_/GFR_cr_ in association with comorbidities and higher mortality in the group.
- Low-GFR_cys_/GFR_cr_ patients might represent those with imminent prerenal acute kidney injury (AKI), as shown by signs of increased urea re-absorption and elevated serum cystatin C (an early indicator of renal endothelial damage) despite low serum creatinine.
- Possible gastrointestinal bleeding, commonly observed in critically ill COVID-19 patients, could contribute to increased serum urea levels, although no significant difference in hematocrit levels was observed between low and high GFR_cys_/GFR_cr_ groups.
- Variations in tubular reabsorption phenotypes might partially explain differences in urea levels. However, given the significant increase in cystatin C (not reabsorbed or secreted in the renal tubule) in low-GFR_cys_/GFR_cr_ patients, other mechanisms likely influence the changes in ratios. Future studies should explore the role of urinary cystatin C and other molecules (electrolytes, glucose, urea, and bicarbonates) normally reabsorbed in proximal tubules to better understand the role of tubular reabsorption in low-GFR_cys_/GFR_cr_ and SPS.

## Conclusions

In conclusion, our study investigated the prevalence and associated factors of low-GFR_cys_/GFR_cr_ in patients with acute, symptomatic SARS-CoV-2 infection. Our findings revealed a significantly higher prevalence of low-GFR_cys_/GFR_cr_ in our cohort compared to previous studies, indicating its potential relevance in the context of acute COVID-19. We observed a strong association between low-GFR_cys_/GFR_cr_ and female sex, older age, and comorbidities like arterial hypertension and diabetes. Moreover, patients with low-GFR_cys_/GFR_cr_ exhibited notably higher mortality rates. We suggest that there are some states that are likely to hide under the SPS, especially in the settings of AKI.

This study offers readers a critical perspective when evaluating similar research, revealing a notable absence of additional diagnostic criteria for SPS. Our results underscore the necessity for more extensive studies, particularly involving larger cohorts.

### Electronic supplementary material

Below is the link to the electronic supplementary material.

Supplementary Material 1

## Data Availability

The dataset supporting the conclusions of this article is available in the Dataverse repository, https://dataverse.rsu.lv/dataset.xhtml?persistentId=doi:10.48510/FK2/GPOBYW.

## Abbreviations

AKI: Acute kidney injury
COPD: Chronic obstructive pulmonary disease
CKD: Chronic kidney disease
eGFR: Estimated glomerular filtration rate
eGFR_creatinine_: Estimated glomerular filtration rates calculated based on creatinine
eGFR_cystatinC_: Estimated glomerular filtration rates calculated based on cystatin C
GBM: Glomerular basal membrane
high-GFR_cys_/GFR_cr_: eGFRcystatinC/eGFRcreatinine ratio > 0.6
IQR: Interquartile range
KDIGO: Kidney Disease:Improving Global Outcomes
low-GFR_cys_/GFR_cr_: eGFRcystatinC/eGFRcreatinine ratio < 0.6
NGAL: Neutrophil gelatinase-associated lipocalin
P/F ratio: Ratio between pO_2_/FiO_2_
RT-PCR: Reverse transcriptase polymerase chain reaction
SARS-CoV-2: Severe acute respiratory syndrome coronavirus 2
SPS: Shrunken Pore Syndrome
