# Supplementary material for: eGFRcystatinC/eGFRcreatinine ratio < 0.6 in patients with SARS-CoV-2 pneumonia: a prospective cohort study
Source: BMC Nephrol. 2023 Sep 13;24:269. doi: 10.1186/s12882-023-03315-x (PMC10500727; doi:10.1186/s12882-023-03315-x)
Supplement: Supplementary file 1 — Supplementary Material 1 [file 12882_2023_3315_MOESM1_ESM.docx]

| Comorbidities, n (%) | Death | | Overall (n=59) | *p*, value |
| --- | --- | --- | --- | --- |
|  | yes (n=15) | no (n=44) |  |  |
| Diabetes | 6 (40.0) | 15 (34.1) | 21 (35.6) | 0.680 |
| Arterial hypertension* | 11 (73.3) | 31 (70.5) | 42 (71.2) | 0.832 |
| Gout* | 1 (6.7) | 2 (4.5) | 3 (5.1) | 0.747 |
| Adiposity | 7 (46.7) | 14 (31.9) | 21 (35.6) | 0.300 |
| Congestive heart failure | 4 (26.7) | 16 (36.4) | 20 (33.9) | 0.493 |
| Gastritis* | 0 (0) | 4 (9.1) | 4 (6.8) | 0.226 |
| Atrial flutter* | 3 (20.0) | 10 (22.7) | 13 (22.0) | 0.826 |
| Liver cirrhosis* | 1 (6.7) | 1 (2.3) | 2 (3.4) | 0.417 |
| Hypothyroidism | 1 (6.7) | 2 (4.5) | 3 (5.1) | 0.747 |

**Additional file 1.** Associations between comorbidities and comorbidities.

*eGFRCreatinine- estimated glomerular filtration rate (Creatinine).*

* Test has cells with count less than expected.

To analyze data Chi-Square test is used.
